# A dPCR-NIPT assay for detections of trisomies 21, 18 and 13 in a single-tube reaction-could it replace serum biochemical tests as a primary maternal plasma screening tool?

**DOI:** 10.1186/s12967-022-03455-y

**Published:** 2022-06-15

**Authors:** Peng Dai, Yanfeng Yang, Ganye Zhao, Zhiqiang Gu, Huanan Ren, Shuang Hu, Ning Liu, Weimeng Jiao, Jinfang Li, Xiangdong Kong

**Affiliations:** 1 The Genetics and Prenatal Diagnosis Center, The Department of Obstetrics and Gynecology, The First Affiliated Hospital of Zhengzhou University, Zhengzhou, 450052 Henan China; 2 Shanghai Tage Biotechnology Co., Ltd, Shanghai, 201201 China; 3 Henan Newbern Medical Technology Co. Ltd, Zhengzhou, 450053 Henan China

**Keywords:** Autosomal aneuploidies, Digital PCR, Non-invasive prenatal test, Serum biochemical tests

## Abstract

**Background:**

The next generation sequencing (NGS) based non-invasive prenatal test (NIPT) has outplayed the traditional serum biochemical tests (SBT) in screen of fetal aneuploidies with a high sensitivity and specificity. However, it has not been widely used as a primary screen tool due to its high cost and the cheaper SBT is still the choice for primary screen even with well-known shortages in sensitivity and specificity. Here, we report a multiplex droplet digital PCR NIPT (dPCR-NIPT) assay that can detect trisomies 21, 18 and 13 (T21, T18 and T13) in a single tube reaction with a better sensitivity and specificity than the SBT and a much cheaper price than the NGS-NIPT.

**Methods:**

In this study, the dPCR-NIPT assay’s non-clinical characteristics were evaluated to verify the cell free fetal DNA (cffDNA) fraction enrichment efficiencies, the target cell free DNA (cfDNA) concentration enrichment, the analytical sensitivity, and the sample quality control on the minimum concentration of cfDNA required for the assay. We validated the clinical performance for this assay by blindly testing 283 clinical maternal plasma samples, including 36 trisomic positive samples, from high risk pregnancies to access its sensitivity and specificity. The cost effectiveness of using the dPCR-NIPT assay as the primary screen tool was also analyzed and compared to that of the existing contingent strategy (CS) using the SBT as the primary screen tool and the strategy of NGS-NIPT as the first-tier screen tool in a simulating situation.

**Results:**

For the non-clinical characteristics, the sample processing reagents could enrich the cffDNA fraction by around 2 folds, and the analytical sensitivity showed that the assay was able to detect trisomies at a cffDNA fraction as low as 5% and the extracted cfDNA concentration as low as 0.2 ng/μL. By testing the 283 clinical samples, the dPCR-NIPT assay demonstrated a detection sensitivity of 100% and a specificity of 95.12%. Compared to the existing CS and the NGS-NIPT as the first-tier screen strategy, dPCR-NIPT assay used as a primary screen tool followed by the NGS-NIPT rescreen is the most economical approach to screen pregnant women for fetal aneuploidies without sacrificing the positive detection rate.

**Conclusion:**

This is the first report on a dPCR-NIPT assay, consisting of all the necessary reagents from sample processing to multiplex dPCR amplification, can detect T21, T18 and T13 in a single tube reaction. The study results reveal that this assay has a sensitivity and specificity superior to the SBT and a cost much lower than the NGS-NIPT. Thus, from both the test performance and the economic benefit points of views, using the dPCR-NIPT assay to replace the SBT as a primary screen tool followed by the NGS-NIPT rescreen would be a better approach than the existing CS for detection of fetal aneuploidies in maternal plasma.

**Supplementary Information:**

The online version contains supplementary material available at 10.1186/s12967-022-03455-y.

## Introduction

Trisomy 21 (T21, Down syndrome), trisomy 18 (T18, Edwards syndrome), and trisomy 13 (T13, Patau syndrome) are the most common autosomal aneuploidies that cause mental retardation and serious congenital defects of newborns [1–3], the prevalence per 10,000 live births is 12.3–17.26, 3.43–4.08 and 1.49–1.68 for T21, T18 and T13, respectively [4–6], and the number would be higher if counting the fetal loss and pregnancy termination after prenatal diagnosis. Among the life birth of infants with T18 or T13, about 50% of them would not survive the first week and only about 10% of them would survive the first year [3]. Whereas 88.9% in 1983 and 87.5% in 2006 of live birth infants with T21 would live to at least 15 and 25 years, respectively [7], and many of them would live to 50 years or older [8]. Even though with a relatively longer life expectancy, other than intellectual disability, individuals with Down Syndrome almost all suffer one or more long term health problems through their lifespan, such as cardiac complications, pulmonary hypertension, hematologic and oncologic disorders, neurodevelopmental disorders, respiratory complications, dysphagia, and others[1, 9]. Recent publications investigated the impacts of COVID-19 disease on individuals with Down syndrome and concluded that the above health conditions might make these individuals at higher risk of SARS-CoV-2 infection or more severe clinical symptoms than their counterparts without Down syndrome [10–12]. Currently, there is no cure for Down’s syndrome, so prenatal detection would provide affected families the options of preparing the birth of a child with Down’s syndrome or terminating the current pregnancy. Conventionally, screen of the fetal aneuploidies is normally done in the first and second trimesters by testing the maternal serum serological makers, typically including pregnancy associated plasma protein A (PAPP-A), human chorionic gonadotropin (hCG), alpha-fetoprotein (AFP), unconjugated estriol (uE3), and Inhibin A. These markers are normally grouped as double, triple, or quadruple assays with or without in combinations with maternal age. These serum biochemical tests (SBT) showed a positive detection rate of 49–76% with 5% false positive rate[13]. The high risk pregnancies identified by the SBT are then referred to chorionic villus sampling (CVS) or amniocentesis (AC) tests that are defined as the gold stand for final diagnosis of the fetal aneuploidies, however, these two invasive procedures are time consuming and may also have up to 1% miscarriage rate[14–16]. Instead of quantitatively and directly measuring the chromosomal dosages related to T21, T13 and T13, the SBT targets surrogate serological markers and indirectly estimates the risks or probabilities if a pregnancy would carry a fetus with aneuploidy. Unavoidably, in addition to a relative low sensitivity, the SBT also have a high false positive rate. In fact, about 95% of the “high risk” pregnancies identified by the SBT were false positive and would unnecessarily undergo through the expensive and stressful invasive CVS or AC test, as well as taking a risk of miscarriage [17, 18].

Since the discovery of cell free fetal DNA (cffDNA) in maternal plasma in 1997[19], several nucleic acid detection technologies (NADT) have, in proof-of-concepts, applied to the noninvasive prenatal test (NIPT) of aneuploidies, such as next generation sequencing (NGS) [20, 21], DNA methylation [22], Matrix-assisted laser desorption/ionization-time of flight mass spectrometry [23], microarray [24], and digital PCR [25]. So far, the NGS is the most successful and recognized NADT used for prenatal screens of fetal aneuploidies, recent mete-analyses indicated that the pooled sensitivity for T21, T18, and T13 were in a range of 99.3–99.4%, 97.4–97.7%, and 90.6–97.5%, respectively, while the pooled specificity was 99.9% for these three trisomies [26–29]. Since its clinical implementations, more affected pregnancies have been identified, meanwhile the number of CVS and AC procedures have dramatically dropped. However, the high overall costs and operational complexities of the NGS-NIPT might hinder its implementation to a wide-spread clinical routine practice. Even though, the NGS-NIPT has been recommended in many counties as the first-tier screen method for fetal aneuploidies screen, majority of pregnant women cannot afford to choose this test because it is currently not universally covered by healthcare systems or private insurances [30, 31]. To effectively balance the benefits and higher cost regarding the NGS-NIPT, many countries select the contingent strategy (CS) that uses the SBT as a primary screen tool and use the NGS-NIPT as a confirmation test for the high risk pregnancies identified by the SBT. The CS showed a better cost-effectiveness when compared to the SBT only strategy and NGS-NIPT as the first-tier test [32–35]. The main advantage of the CS is its ability to remarkably reduce the unnecessary invasive procedures, but its positive detection rate would in principle not be much better than the SBT, since all high risk pregnancies are still determined by the SBT at the forefront of this strategy [35]. However, if the SBT can be replaced by a NADT assay with a higher sensitivity similar to the NGS-NIPT and a lower price close to the SBT, the upgraded CS would currently be a preferred solution to screen all pregnancies. Digital PCR (dPCR) is one of the most suitable NADT platforms for replacing the SBT due to its incomparable capability in absolute quantification of nucleic acids [36]. In the dPCR process, the PCR reaction mix containing targets of interests is randomly distributed into tens of thousands independent partitions and each partition contains one (or few) or no target molecule. After PCR, the proportion of positive partitions is applied to accurately quantify the target concentration using Poisson’s statistics [36–38]. There are quite a few publications demonstrated the usefulness of dPCR technology in detection of fetal T21 from variety of angles, some used the earlier versions of dPCR platforms or invasive CVS/AS samples to prove the concept [25, 39, 40], while others evaluated the feasibilities in detections of fetal T21 and T18 in maternal plasma samples [41–44].

Here we describe the performances of a multiplex dPCR-NIPT technique for simultaneous detections of fetal T21, T18 or T13 in a single tube reaction on a droplet dPCR platform, as well as its preliminary clinical performance in testing real clinical samples in a clinical environment. By the best of our knowledge at the time this manuscript being prepared, this is the first report to demonstrate that a full dPCR-NIPT technique, consisting of sample processing, cffDNA fraction enriching, the target cell free DNA (cfDNA) concentration enrichments, and dPCR amplification reagents, can simultaneously detect fetal T21, T18 or T13 in maternal plasma samples in a single reaction. The justifications of replacing the SBT with this dPCR-NIPT assay for the upgraded CS as mentioned above are also discussed.

## Materials and methods

### Study design and sample information

To evaluate how this dPCR-NIPT assay would perform in a real clinical setting, we performed the entire assay process by testing true clinical samples in the First Affiliated Hospital of Zhengzhou University. A total of 453 high risk pregnant women with singleton pregnancy were recruited for this study and their high risk statuses were identified by the hospital’s routine first and/or second trimester screens (including the SBT, and/or ultrasound) for fetal aneuploidies. Some of pregnancies in this population directly selected or prescribed by their doctors for the NGS-NIPT screen, and, except a few, all the high risk pregnancies identified by the SBT and/or ultrasound were further rescreened by the NGS-NIPT method to confirm the high risk results and filter out the false positives. The NGS-NIPT high risk pregnancies were finally diagnosed by the invasive CVS/AC procedures. A separate aliquot (ca. 2.5 mL) of each maternal plasma samples was kept a side at -20ºC for this dPCR-NIPT test. Of the 453 samples, 170 (including 2 T21, 1 T18 and 1 T13 samples) were randomly picked and used as training set for onsite training and assay critical procedure validations, as well as for establishing euploid baseline cut-off value for this assay. The clinical characteristics for the training set samples are shown in Additional file 1: Table S1. The remaining 283 samples (including 25 T21, 10 T18 and 1 T13 samples), served as a testing set, were screened by the dPCR-NIPT assay in a blind manner. For this set of samples, the median gestational age was 17 weeks with a range of 12–36 weeks, while the median maternal age in this set of pregnant women was 30 years with a range of 17–44 years. The routine screen procedure identified 30 (83.3%) and 35 (97.2%) of the 36 trisomy cases before the maternal age of ≤ 34 years and the gestational age group of ≤ 20 weeks, respectively, indicating the importance of timely screen in the first and second trimester regardless of maternal age (Table 1).

**Table 1:** Testing set sample information

Clinical samples sorted by	T21	T18	T13	Normal	Total
Maternal age (Years)					
≤ 24	4	1	0	27	32
25–34	17	7	1	169	194
≥ 35	4	2	0	51	57
Gestational age (Weeks)					
12–15	17	9	1	28	55
16–20	7	1	0	191	199
21–25	0	0	0	17	17
≥ 26	1	0	0	11	12
Screen method					
SBT HR (Cut-off at 1/270)	5	0	0	38	43
SBT IR (Cut-off at 1/271–1/1000)	3	0	0	63	66
Abnormal Ultrasound Results	15	9	1	23	48
Adv. Maternal Age (≥ 35 Years)	1	0	0	47	48
Direct NGS-NIPT	1	1	0	76	78
Total	25	10	1	247	283

### Sample preparation

The median or average cffDNA fraction in maternal plasma is generally considered around 10% in the first trimester and would dramatically increase to about 20% in the third trimester [45–47], while that for female fetuses is relatively higher than male fetuses in the same gestational weeks [46]. Since the screens for fetal aneuploidies usually start in the first trimester when the cffDNA proportion is relatively low, therefore in this study, we used the magnetic bead based Extraction and cffDNA Enrichment Kit (the Tage SMP Prep Kit, for research use only) provided by Tage Biotechnology Co., Ltd. (Tage Biotech, Shenzhen, China) for maternal plasma cfDNA extraction and cffDNA enrichment. As a part of the validation and training activities, the cffDNA fraction enrichment function of the SMP Prep Kit was evaluated by processing and dPCR testing 10 euploid plasma samples from pregnancies with male fetuses. For formal sample process for the testing set samples, 2.5 mL of each saved plasma aliquots was used for the cfDNA extraction and cffDNA enrichment on a semi-automatic PF32A Nucleic Acid Extraction System (Genefaster Co., Ltd., Shanghai, China), and the isolated cfDNA with its cffDNA portion enriched was finally eluted with 40 μL of TE-buffer and stored at − 20 °C or colder until use. After extraction, the cfDNA concentration was measured using Qubit 3 Fluorometer and Qubit DS DNA and BR Array Kit (Thermo Fisher, USA) for each isolated sample and only samples with a DNA concentration ≥ 0.2 ng/μL was qualified for the dPCR-NIPT assay.

### Target specific primer and probe designs

To increase the assay sensitivity, multiplex dPCR was applied to quantitate Chromosomes 21, Chromosome 18, and Chromosome 13 (Chr21, Chr18, and Chr13). Candidate primers and probes were chosen for each of the three chromosomes by searching the National Center for Biotechnology Information’s (NCBI) database with its online tools of Nucleotide-BLAST and Primer-BLAST provided on its website (https://www.ncbi.nlm.nih.gov). These candidate sequences were analyzed to ensure no primer-dimmers and hairpin-structures exist with an online tool provided by iGeneTech (https://mfeprimer3.igenetech.com). The primers and probes passed above selection steps were sent to ThermoFisher Scientific (https://www.thermofisher.cn) for syntheses and the detection probes of Chr21, Chr18, and Chr13 were labeled with FAM, VIC, and Cy5 fluorophores, respectively, to differentiate the amplification signals. After a series of evaluation experiments, 10 sets primers and probes for each of the three chromosomes (Chr21, Chr18, and Chr13) were selected for this dPCR-NIPT assay and the sequence information are presented in Additional file 1: Table S2.

### General description of the dPCR-NIPT assay

Since it is generally accepted in principle that there is no co-existent of two aneuploidies in a singleton pregnancy regarding T21, T18 and T13, thus, when one of these three chromosomes is investigated for fetal aneuploidy, the other two would serve as euploidy references in chromosome copy number (CCN) comparisons. Based on this concept, we designed the dPCR-NIPT assay for detecting fetal aneuploidies by three major steps: (1) measuring the CCN for Chr21, Chr18, and Chr13 by the dPCR-NIPT assay in a single tube reaction, while the CCN for each chromosome is automatically calculated by the instrument’s software based on Eq. 1; (2) calculating the CCN ratios of Chr21/Chr18, Chr21/Chr13 and Chr18/Chr13 (R_21/18_, R_21/13_, and R_18/13_), separately, for the sample using Eq. 2; and (3) calculating the Z-score value for R_21/18_, R_21/13_, and R_18/13_ (Z_21/18_, Z_21/13_, and Z_18/13_) for the sample using Eq. 3.

1 $$\mathrm{CCN}/\mathrm{\mu L }= - \frac{Ln \left(1-\frac{d}{n}\right)}{V}$$

where *n* is the total number of effective droplets in a dPCR reaction, *d* is the number of positive droplets, and V is the volume of a single droplet (μL).

2 $${R}_{ChrT/ChrR}= \frac{CCN\,of\,ChrT}{CCN\,of\,ChrR}$$

where, R_ChrT/ChrR_ is the CCN ration between the chromosome being tested (ChrT) and the reference chromosome (ChrR) from the same sample in the same dPCR assay. When R_21/18_ (or R_21/13_, or R_18/13_) is calculated. ChrT represents Chr21 (or Chr21, or Chr18) and ChrR represents Chr18 (or Chr13, or Chr13), respectively.

3 $${Z}_{ChrT/ChrR}= \frac{{Sample\,R}_{ChrT/ChrR} - BL Mean {R}_{ChrT/ChrR} }{SD\,of\,BL\,Mean {R}_{ChrT/ChrR}}$$

where, Z_ChrT/ChrR_ is the Z score of the R_ChrT/ChrR_ of a sample being tested, BL Mean R_ChrT/ChrR_ is the mean R_ChrT/ChrR_ of a euploid pregnant population that serves as a baseline (BL) R_ChrT/ChrR_ for a cut-off value determination, and SD is the standard deviation of BL Mean R_ChrT/ChrR_. When Z_21/18_ (or Z_21/13_, or Z_18/13_) is calculated, the Sample R_ChrT/ChrR_ is the R_21/18_ (or R_21/13_, or R_18/13_) of a sample being tested and the BL Mean R_ChrT/ChrR_ is the mean R_21/18_ (or R_21/13_, or R_18/13_) of a euploid pregnant population.

### dPCR instrument

For the dPCR-NIPT assay in this study, we used BioDigital-QING dPCR™ system (Shanghai Turtle Technology Co., Ltd., Shanghai, China, http://www.turtle-tech.cn), which consists of three separate instruments: BioDigital-QING Loader™ for generating one layer of water-in-chamber droplets inside a microfluidic chip (Additional file 1: Fig. S1), BioDigital-QING Cycler™ for PCR amplification, and BioDigital-QING Imager™ with four fluorescence detection channels for positive and negative signal captures (Additional file 1: Fig. S2). A unique advantage of the microfluidic chip is that it uses curable oil to not only partition PCR reaction mix into chambers but also further stabilize the droplets and prevent sample cross-contamination. This system needs a 30 μL of starting PCR reaction mix to form around 20,000 microdroplets within 2 min and less than 3 h to complete the whole dPCR process with an effective droplet ratio of 95% [48, 49]. The current throughput for this system is 24 reactions per batch of dPCR run, however, when one more PCR machine is added into this system, the throughput can reach up to 96 dPCR reactions within 10 h.

### dPCR reactions

#### Target cfDNA enrichment by pre-amplification

For the testing set samples, the cfDNA concentration isolated from a 2.5 mL of maternal plasma was relatively low with an average and median value of 0.36 and 0.34 ng/μL (range 0.20–0.87 ng/μL), respectively. Thus, a pre-amplification (PA) step is required to boost the target cfDNA concentrations for accurate CCN quantification.

For target enrichment, 10 μL of isolated cfDNA from each sample was mixed with 5 μL of pre-amplification master mix (PA-MMX) (Tage Biotech, for research use only) and amplified with 6 cycles of PCR with the following cycling conditions: 95 °C for 10 min, 6 cycles of 95 °C for 30 s and 60 °C for 30 s. The PA-MMX contains 10 primer pairs each for Chr21, Chr18, and Chr13 for specifically enriching the designated cfDNA targets on the three chromosomes. Ten (10) μL of the enriched target cfDNA was used in dPCR test after a 6.25-fold dilution, which was to ensure the positive droplet rate fallen within the 30–80% range for more actual CCN measurements. We evaluated the efficacy of cfDNA specific target enrichments by testing 16 normal maternal plasma samples without and with PA.

#### dPCR-NIPT assay operations and assessments

In a 30 μL multiplex reaction, we added 10 μL of enriched and diluted cfDNA targets into 20 μL of dPCR master mix (MMX) containing the same 30 primer pairs as those in PA-MMX, plus the corresponding detection probes for Chr21, Chr18, and Chr13, the resulted 30 μL dPCR reaction mix was tested by using the Biodigital-Qing dPCR™ system according to the manufacturer’s instruction. The dPCR cycling conditions was as follows: 95 °C for 10 min, 45 cycles of 95 °C for 30 s and 60 °C for 30 s. The total CCN (presented as copies/μL) cumulated from the 10 amplification sequences for Chr21, Chr18, or Chr13 was automatically calculated by the analysis software of the dPCR system according to the Poisson distribution, then, R_21/18_, R_21/13_, and R_18/13_, as well as Z_21/18_, Z_21/13_, and Z_18/13_ were sequentially calculated as described above.

We accessed the dPCR-NIPT assay’s analytical sensitive regarding the effect of cffDNA fraction in the maternal plasma on the trisomy detections by testing a panel of artificial T21, T18 or T13 samples. Appropriate amounts of 10 synthetic Chr21, Chr18 or Chr13 targets (simulating cffDNA) matching to the 10 amplification sequences on its corresponding chromosome were added to a purified maternal cfDNA (50 copies/μL) to a final “cffDNA” fraction of 0%, 3%, 5%, 10%, or 15%, 10 μL of the artificial trisomy samples was tested by the dPCR-NIPT assay in 8 replicates.

#### dPCR-NIPT assay of the testing set samples

The 283 plasma samples of the testing set were tested by the dPCR-NIPT assay following the manufacturer’s instructions, throughout the testing processes the samples’ true aneuploidy statuses were kept unknown to all operators performing the dPCR test until all sample tests completed and conclusions made. Then, the dPCR-NIPT test results were compared to CVS/AS confirmed results for these samples. Whenever possible, the test results of the dPCR-NIPT were also compared to those of the SBT and NGS-NIPT to evaluate the dPCR-NIPT assay’s clinical performances.

## Results

### cffDNA enrichment efficiency

To assess the cffDNA enrichment efficiency of the SMP Prep Kit, 10 maternal plasma samples of normal pregnancies with male fetuses were processed with this kit and their cfDNA and cffDNA copy numbers were directly measured with dPCR without PA at cfDNA extraction and cffDNA enrichment stages. The results indicated that the SMP Prep Kit was capable to increase the cffDNA fraction by 1.73–2.42 folds (Table 2), which would enhance the dPCR-NIPT assay’s capability in fetal aneuploidy detections.

**Table 2:** cffDNA enrichment efficiency

SMP#	cfDNA Extraction Stage			cffDNA Enrichment Stage			
	cfDNA (Copies)	cffDNA (Copies)	cffDNA (%)	cfDNA (Copies)	cffDNA (Copies)	cffDNA (%)	Enrichment (X)
1	351	14	3.90	190	15	8.05	2.07
2	579	33	5.70	329	37	11.32	1.99
3	462	26	5.57	283	29	10.25	1.84
4	372	21	5.52	218	24	11.12	2.01
5	407	21	5.20	239	25	10.36	1.99
6	434	41	9.45	286	47	16.35	1.73
7	359	35	9.75	222	38	17.12	1.76
8	564	33	5.76	325	39	11.92	2.07
9	467	23	4.98	247	26	10.52	2.11
10	478	23	4.81	219	26	11.64	2.42

The copy numbers for cfDNA and cffDNA were shown as copies/reaction and directly measured with the dPCR assay without pre-amplification. X, indicating times of enrichment

### Impact of pre-amplification on CCN ratio

**Fig. 1:**
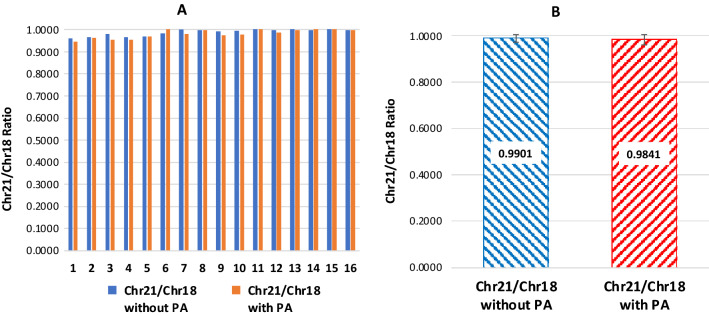
The copy number ratio (R) between Chromosome 21 (Chr21) and Chromosome 18 (Chr18) (R_21/18_) without and with 7 cycles of pre-amplification (PA) before the dPCR-NIPT assay, in which 8 µL of 12.5 fold-diluted PA product was used. **A** R_21/18_ of 16 individual euploid plasma samples without (blue solid bars) and with (red solid bars) PA. **B** Mean R_21/18_ of the 16 euploid samples without (blue right slash lines) and with (red left slash lines) PA valued at 0.9901 with a SD of 0.0175 and 0.9841 with a SD of 0.0225, respectively

To evaluate whether introducing the PA step in this dPCR-NIPT would influence the CCN ratios, which is the most critical point for a dPCR based NIPT test, we used the R_21/18_ as an example to validate this approach. The cfDNA was extracted from each of 16 euploid plasma samples and tested by dPCR test both directly and after 7 cycles of PA, whereas the PA product was diluted 12.5 folds before being tested by dPCR. The R_21/18_ without and with PA for each of the 16 samples was extremely close with a mean paired difference of 1.24% (range from 0.08% to 2.52%, data not shown), while the mean R_21/18_ (SD) of the 16 samples without and with PA were 0.9901 (0.0175) and 0.9841 (0.0225), respective, which are statistically no difference (*P* > 0.4) (Fig. 1). We concluded that PA had no significant impact on the CCN, therefor, decided to incorporate the PA step in the dPCR-NIPT assay.

### Analytic sensitivity of the dPCR-NIPT assay

**Fig. 2:**
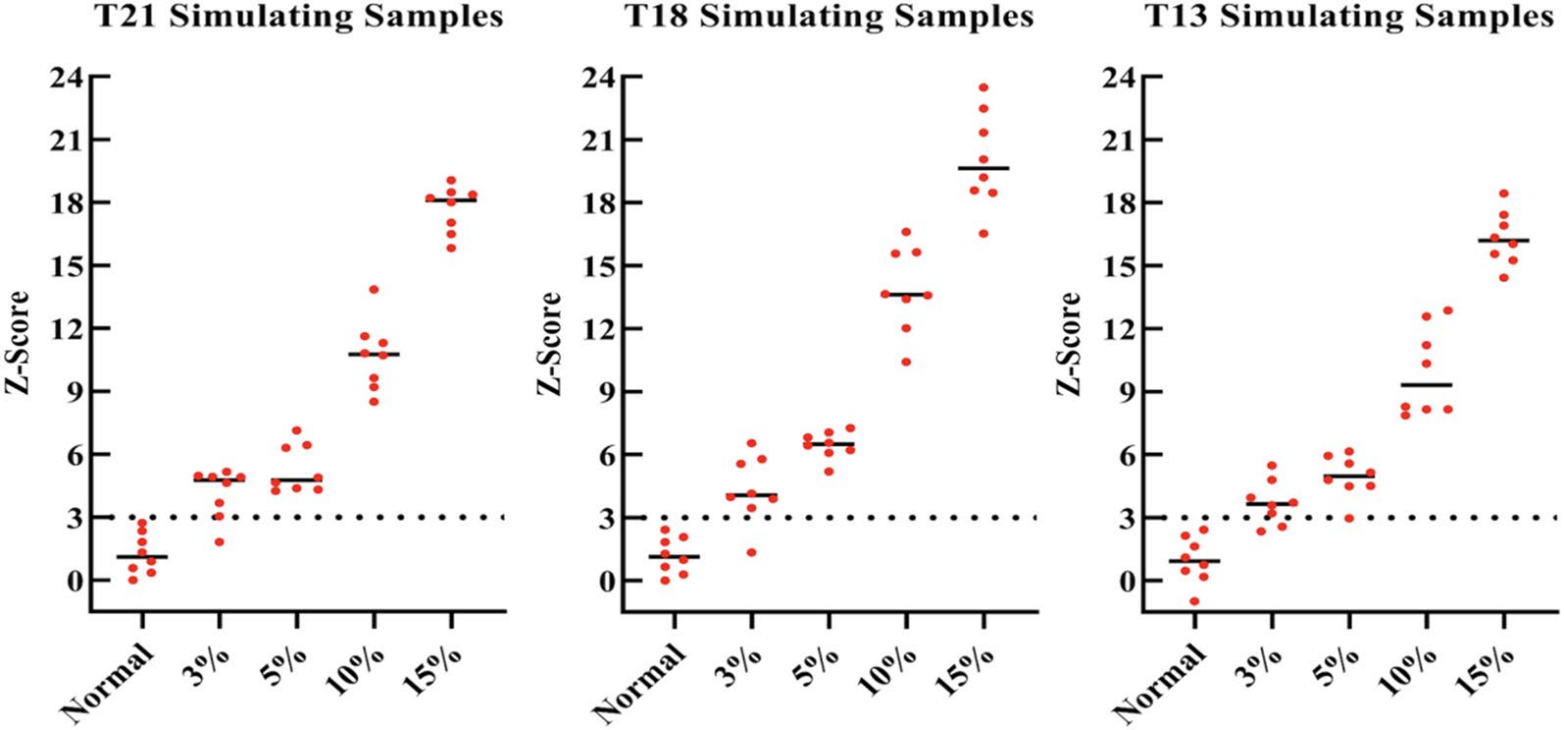
A panel of simulating T21, T18 and T13 samples were prepared, separately, by adding synthetic Chr21, Chr18 or Chr13 specific targets (simulating as trisomy cffDNA) into maternal cfDNA (50 copies/μL) at a final fraction of 0% (normal), 3%, 5%, 10%, and 15%, 10 uL each of the simulating trisomy samples was tested in 8 replicates by the dPCR-NIPT assay. Z-scores were calculated and plugged against the "cffDNA" fractions. The long horizontal dotted line indicates the cut-off value with a Z-score at 3 and the short solid line in each data group represents the median Z-score value

We evaluated the analytical sensitivity of the dPCR-NIPT assay by testing a panel of simulating trisomy samples and the results were shown in Fig. 2. Against a Z-score cut-off of 3, the positive detection rate at 3% of cffDNA fraction was 87.5% (7/8) for T21, 75% (6/8) for T18, and 75% (6/8) for T13, all much lower than our desired sensitivity of ≥ 95%. Thus, with the dPCR instrument (about 20,000 droplets available) used in this study, the lowest cffDNA fraction that a trisomy could reliably be detected was at 5%, since the positive detection rate was 100% (8/8) for all trisomies at this concentration. As discussed later in this report, to detect a trisomy at a cffDNA fraction lower than 5% would need a much higher number of droplets than 20,000. Nevertheless, the simulating test results implied that the dPCR-NIPT assay can potentially be used for detection of trisomies at a cffDNA fraction as low as 5% with 20,000 droplets.

### Readiness for the clinical sample testing

There are three main challenges the dPCR-NIPT assay need to overcome before this assay can be implemented in clinical sample testing: the relatively low cffDNA fractions in maternal plasma, generally insufficient total cfDNA amount due to limited available sample volume, and the number of droplets provided by a dPCR system. We resolved the first two sample related challenges by creating and incorporating the general cffDNA fraction enrichment and target cfDNA PA technologies to this dPCR-NIPT assay (Table 2 and Fig. 1). Of cause, the natural cffDNA and cfDNA characteristics of the maternal plasma samples are also crucial, we examined their distributions for the 283 testing-set samples categorized by the maternal age and gestational age (Table 3). When linked to the maternal ages, the mean and median cffDNA fractions for the three maternal age groups were all in tight range of 10.99–13.37% and 9.55–13.09%, respectively. However, when analyzed based on the gestational weeks, we noticed that the mean and median cffDNA fractions positively correlated to the gestational weeks, at 10.91% and 9.76% for the 12–15 week group, 11.94% and 11.24% for the 16–20 week group, 12.33% and 12.19% for the 21–25 week group, and 17.84% and 17.41% for the ≥ 26 week group, respectively. The mean and median cffDNA fractions in the ≥ 26 gestational week group were statistically different from the other three gestational week groups (*P* < 0.0001), which agrees with the reported results[46]. In contrast, our data indicated that the cfDNA concentration seemed not sensitive to either the maternal age and the gestational week, since the mean (range within 0.32–0.37 ng/μL) and median (range within 0.31–0.37 ng/μL) values were very similar among these groups (*P* > 0.8), except the ≥ 26 gestational week group which was statistically different from the other groups (*P* < 0.05). In general, the cffDNA fraction for more than 99% of maternal plasma samples would be ≥ 4% with a median value around 10% [45–47, 50], we believe that both the cffDNA fraction and the cfDNA concentration in maternal plasma would be sufficient to the dPCR-NIPT assay, which is armed with the unique capabilities of cffDNA enrichment, cfDNA PA and multiplex detection, for fetal aneuploidies screens.

**Table 3:** cffDNA fraction and cfDNA concentration information

Categorized by	Number	cffDNA(%)			cfDNA(ng/uL)		
		Mean	Median	Range	Mean	Median	Range
Mat. age (Years)							
≤ 24	32	13.40	13.24	6.41–21.03	0.35	0.33	0.22–0.72
25–34	194	12.10	11.21	3.10–34.64	0.36	0.34	0.20–0.71
≥ 35	57	10.95	9.57	3.72–26.22	0.36	0.35	0.24–0.87
Gest. age (Weeks)							
12–15	55	11.43	10.28	4.09–28.59	0.37	0.35	0.20–0.71
16–20	199	11.89	11.26	3.10–31.21	0.36	0.35	0.22–0.87
21–25	17	11.94	10.87	4.87–21.24	0.32	0.31	0.20–0.50
≥ 26	12	18.88	17.79	4.62–34.64	0.41	0.37	0.23–0.72
Total	283	11.97	11.06	3.10–34.64	0.36	0.34	0.20–0.87

As for the dPCR system, we calculated the minimum number of droplets required for the NIPT purpose under certain conditions according to the mathematic formulas (Eqs. 4 and 5) published [39]. By resolving Y of Eq. 4 and bringing it to Eq. 5, we could obtain Eq. 6, where N estimates the number of droplets required, ε represents cffDNA fraction in the sample, k indicates the number of standard deviations (SD), and Y/N is the positive droplet rate (PDR) in a dPCR reaction. For example, when cffDNA fraction is at 5% (0.05) and PDR is 30% (0.3), we need about 18,000 droplets or PCR reactions to distinguish an aneuploidy pregnancy from a noise of euploid pregnancies with a 95% (1.96 times of SD) confidence. The calculated result is very similar to those of published [39, 51]. The number of droplets required for a dPCR reaction for different PDR and cffDNA fraction combinations was also estimated by using Eq. 6 with a fixed SD at 1.96 (Additional file 1: Table S3). When a cffDNA fraction is ≤ 5%, and PDR ≤ 30%, 20,000 droplets is not sufficient to detect trisomies in a maternal plasma sample with a 95% confidence.

4 $$\mathrm{k}= \frac{\upvarepsilon \sqrt{Y}}{2+\upvarepsilon }$$

5 $$\mathrm{Y}= \frac{\mathrm{log}(1-\frac{\mathrm{Y}}{\mathrm{N}})}{\mathrm{log}(1-\frac{1}{N})}$$

6 $$\mathrm{N}= \frac{1}{1-{10}^{ ( \frac{\mathrm{log}\left(1-\frac{\mathrm{Y}}{\mathrm{N}}\right){\upvarepsilon }^{2}}{(4+4\upvarepsilon +{\upvarepsilon }^{2}){k}^{2}} )}}$$

The BioDigital-QING dPCR™ system used in this study could reliably generate about 20,000 droplets with above 95% effectiveness, meaning a minimum of 19,000 effective droplets routinely available for a dPCR assay. It seemed that the third challenge was also resolved, allowing us to proceed the clinical sample tests.

### Determination of the minimum cfDNA concentration required for maternal plasma samples

**Fig. 3:**
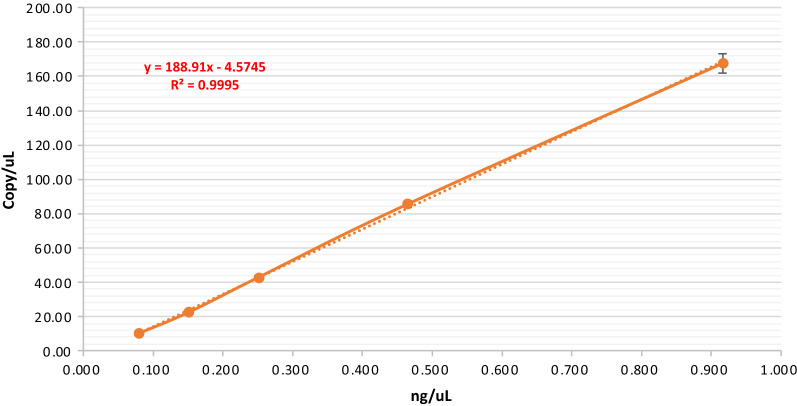
Standard curve for ng/uL and c/uL conversions. The solid line represents the experimental data, and the dashed line is the trendline generated by Excel's trendline tool. The formular is associated with the trendline and used for calculating c/μL from ng/μL. c/μL, copies/μL

Under the conditions mentioned above, at least 30% positive droplets are required to detect aneuploidy pregnancies, which mean a maternal plasma sample should have sufficient cfDNA to provide enough target molecules for the dPCR assay. Thus, we needed to set up a benchmark of minimum cfDNA concentration required for plasma samples to be tested by the dPCR-NIPT assay. It is not practical to measure cfDNA copy number concentration (c/μL) on a routine basis, however, to determine the cfDNA weight concentration (ng/μL) with a fluorometer is convenient. We used Qubit 3 Fluorometer to measure cfDNA concentration (ng/μL) and established a standard curve to convert ng/μL to c/μL (Fig. 3). A cfDNA extracted from a normal maternal plasma sample was serially diluted and the ng/μL concentration at each dilution level was measured before tested by the dPCR assay in 4 replicates. The c/μL concentrations at each dilution level were calculated and plugged against the ng/μL for generating the standard curve, which illustrated a strong linear correlation between the two types of concentrations with a R^2^ value of 0.9995 (Fig. 3).

**Fig. 4:**
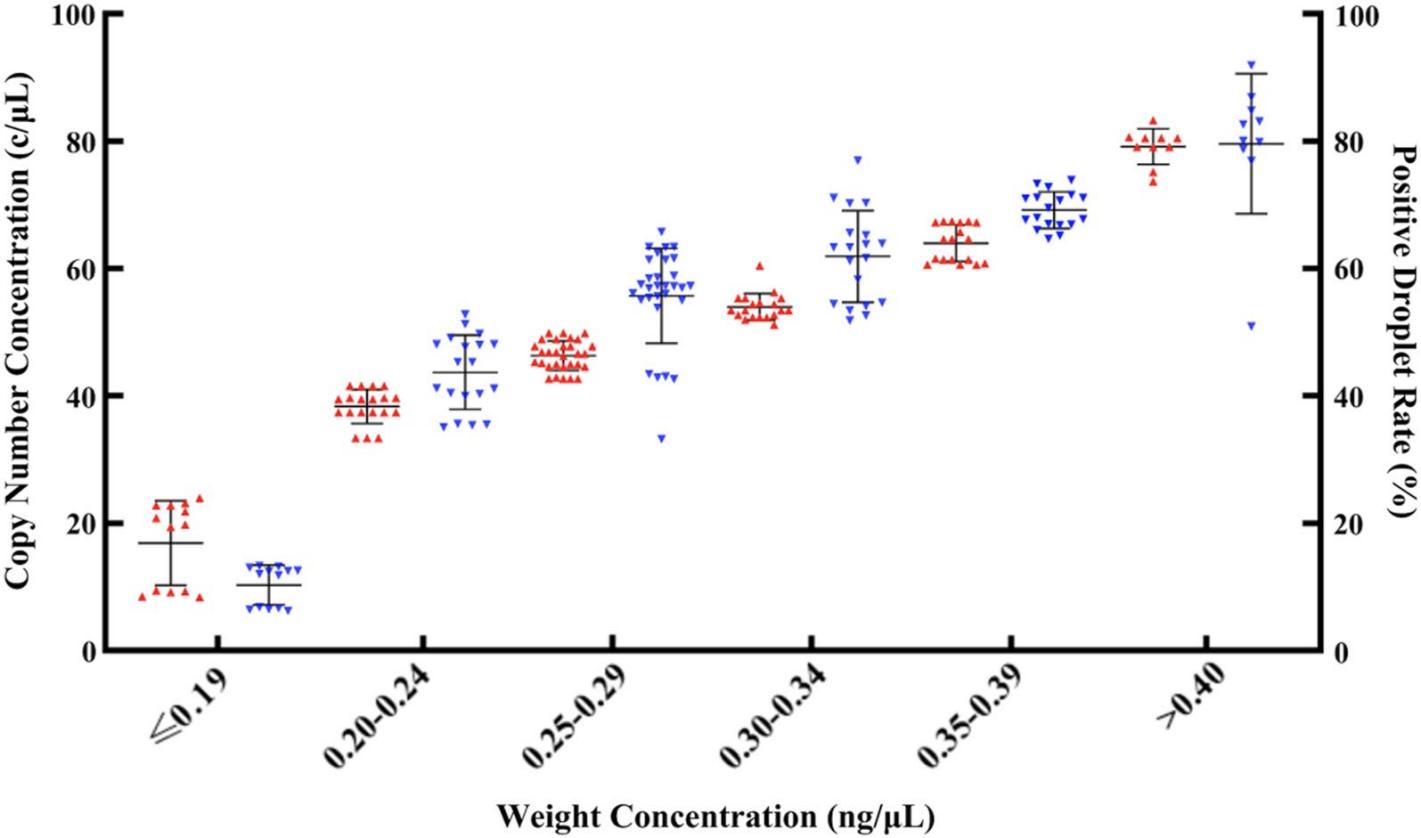
Weight concentration (ng/μL) was measured by Qubit Fluorometer for each sample, and then converted to copy number concentration (c/μL, red upward triangles) with the formula of y = 188.91x−4.5745, where y is c/μL, x is ng/μL. After dPCR assay, the corresponding positive droplet rate (%, blue downward triangles) for each sample was recorded and shown in this figure. The three short lines in each data group represent mean ± 1SD

We evaluated 103 maternal plasma samples of euploid pregnancies to validate the accuracy of the standard curve for concentration unit conversion and the minimum cfDNA concentration needed to reach 30% PDR. The samples were extracted with the Tage SMP Prep Kit, their ng/μL concentrations were measured and converted to c/μL concentration by using the equation of y = 188.91x-4.5745 from Fig. 3, where y is the c/μL concentration and x is the ng/μL concentration. The PDR of each sample was recorded after the dPCR assay and summarized in Fig. 4. When grouped in a narrow weight concentration range with an interval of 0.05 ng/μL, the range of converted c/μL concentrations of each group was relatively tight with a small SD value and no overlaps between the adjacent groups. Meanwhile, the range of the PDR of each group is widespread with obvious overlaps between neighboring groups. Nevertheless, the mean and median of c/μL concentration and of the PDR were positively corresponding to the ng/μL concentrations and statistically different (*P* < 0.05) from group to group. The results reveals that the all samples with a ng/μL concentration ≥ 0.2 had a PDR above 30%, thus, we set the benchmark of minimum cfDNA concentration required right at 0.2 ng/μL for plasma samples to be tested by the dPCR-NIPT assay.

### The dPCR-NIPT assay of the testing set plasma samples

**Fig. 5:**
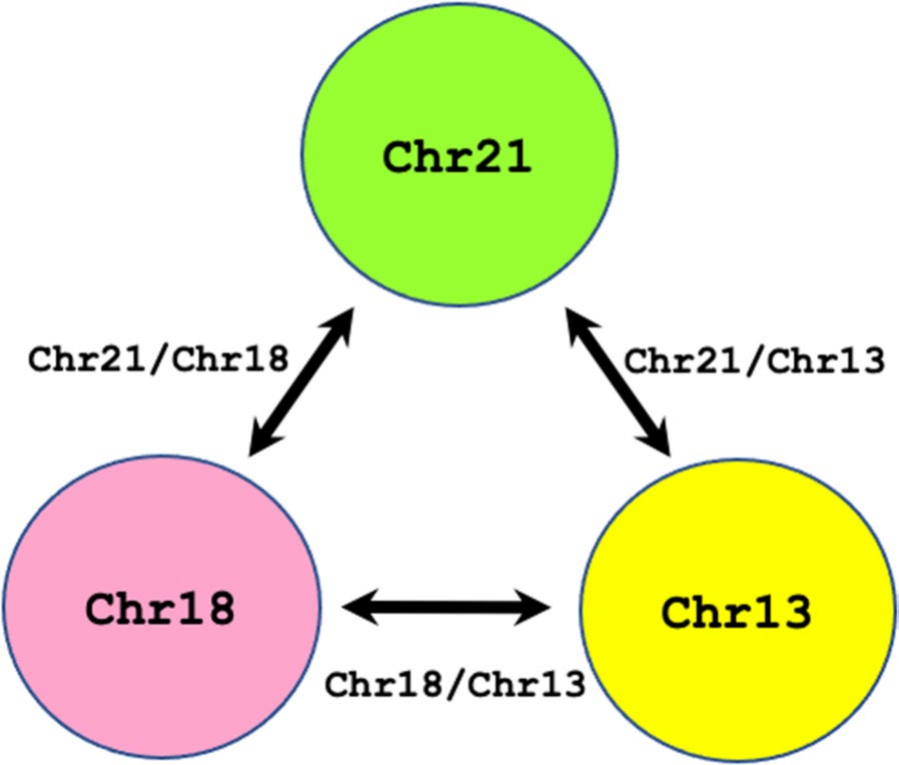
For detecting aneuploidy pregnancies, Chromosomes 21, 18 and 13 (Chr21, Chr18 and Chr13) were used as both the targeting and reference chromosomes. After the dPCR-NIPT test, R_21/18_, R_21/13_ and R_18/13_, as well as the corresponding Z_21/18_, Z_21/13_ and Z_18/13_ for each sample were calculated

Before examining the testing set samples, we established the assay strategies and determined the cut-off criteria. The dPCR-NIPT assay uses Chr21, Chr18 and Chr13 both as the targeting chromosomes and as the reference chromosomes (Fig. 5). When a sample was tested, its Z_21/18_, Z_21/13_ and Z_18/13_ values would be evaluated against the Z-score cut-off value and the aneuploidy status calling algorithm (Table 4). With the dPCR system used in this study and the calculation for the number of droplets required under the conditions described above, we determined to set a Z-score cut-off criterion at 1.65, indicating a theoretical 95% test confidence. A sample with a Z_21/18_ (or Z_21/13_ or Z_18/13_) ≥ 1.65 is called T21 (or T21 or T18) positive, a sample with a Z_21/18_ (or Z_21/13_ or Z_18/13_) ≤ -1.65 is called T18 (or T13 or T13) positive, and a sample with a Z_21/18_ (or Z_21/13_ or Z_18/13_) < 1.65 and > -1.65 is called trisomic negative or low risk.

**Table 4:** Calling algorithm for sample test results

Z-score	Aneuploidy risk			
	Z ≥ 1.65	Z ≤ −1.65	1.65 > Z > − 1.65	
Z_21/18_	T21	T18	Low risk	
Z_21/13_	T21	T13	Low risk	
Z_18/13_	T18	T13	Low risk	
Result scenario	Chromosome pair calls			Final conclusion
	Z_21/18_	Z_21/13_	Z_18/13_	
1	T21	T21	Low risk	T21
2	T21	Low risk	Low risk	T21
3	Low risk	T21	Low risk	T21
4	T18	Low risk	T18	T18
5	T18	Low risk	Low risk	T18
6	Low risk	Low risk	T18	T18
7	Low risk	T13	T13	T13
8	Low risk	T13	Low risk	T13
9	Low risk	Low risk	T13	T13
10	Low risk	Low risk	Low risk	Low risk
11*	Any 2 conflict trisomic calls			Re-test

The calling algorithm for sample test results: the top half portion tabulates the "aneuploidy risk” calls for Z_21/18_, Z_21/13_ and Z_18/13_ of a testing sample, and the lower half portion demonstrates 11 possible scenarios of combined 3 Z-score calls for a sample's test results and the final conclusion for the sample's aneuploidy status

*For Scenario 11, if there are any 2 conflict trisomic calls (for example, Z_21/18_ calls T21 and Z_18/13_ calls T13) for a sample, this sample must be re-tested: (1) if re-test results fall into one of the first 10 scenarios, this sample’s final result should be call according to that scenario; (2) if re-test results fall into Scenario 11 again regardless of any two conflict calls, this sample’s final result should be a no-call

We then test the 283 maternal plasma samples of the testing set, in a blind manner, according to the assay procedures provided by Tage Biotech, briefly, 2.5 mL of plasma sample was processed with the Tage SMP prep kit on Genefaster’s PF32A Automated Nucleic Acid Extraction System with a final elution volume of 40 μL, the purified cfDNA concentration was measured by Qubit 3 Fluorometer (all samples were ≥ 0.2 ng/μL), 10 μL was used for PA (6 cycles), and 10 μL of the PA product was used in the dPCR-NIPT assay after a 6.25-fold dilution. We calculated the CCNs of Chr21, Chr18 and Chr13, the R_21/18_, R_21/13_ and R_18/13_ values, and the corresponding Z_21/18_, Z_21/13_ and Z_18/13_ values for each sample as described above and determined the aneuploidy status for each sample tested according to the calling algorithm tabulated in Table 4.

Of the 283 samples, 8 (2.83%) fell into Scenario 11 and went through the re-test processes with the dPCR-NIPT assay according to the calling algorism (Table 4): 7 of them fell into Scenario 10 (Low Risk) and 1 sample fell into Scenario 11 (No-call). This sample with a “no-call” result was excluded from further data analyses in this study. For the remaining 282 plasma samples, 32, 10, 6, and 234 were identified as T21, T18, T13, and low-risk, respectively, by the dPCR-NIPT assay. Then these initial results of the dPCR-NIPT assay were directly compared to those of karyotypical diagnostics tests (CVS/AC) which are the gold standards. Because most of the “high risk” pregnancies originally identified by the SBT were redefined by the NGS-NIPT test as true low risk and did not undergo CVS/AC tests, thus the dPCR-NIPT assay results for the samples from these women were directly compared to the NGS test results. Based on the samples’ true aneuploid statuses determined by CVS/AC and NGS-NIPT, the dPCR-NIPT assay actually detected 25 of 25 T21 with 7 false positives, 9 of 10 T18 with 1 false positive and 1 false negative, 1 of 1 T13 with 5 false positives (Table 5), indicating a straightforward detection sensitivity and specificity of 100% (25/25) and 97.28% (250/257) for T21, 90% (9/10) and 99.63% (271/272) for T18, and 100% (1/1) and 98.21% (276/281) for T13. Of the 10 true T18 positive samples, 1 was repeatedly identified as T21 positive by the dPCR-NIPT assay. We suspected two possible causes to this conflict result. One might be related to confined placental mosaicism (CPM), because cffDNA is mainly derived from the cytotrophoblasts of chorionic villi in placenta, which is not always representative for the fetus [52]. Unfortunately, we did not get the placental tissue to verify whether CMP occurred to this case. The other might be related to the microheterogeneities of the cfDNA target sequences presented in these samples, a phenomenon associates with the apoptosis processes of both maternal and fetal cells, which leads to the concentration of the cfDNA fragments unequally presented in the maternal plasma [53]. Nevertheless, this sample had to be unfavorably counted as a T18 false negative and a T21 false positive, separately, for the dPCR-NIPT assay’s T18 and T21 evaluations. Since the dPCR-NIPT assay is intentionally used for NIPT screens of fetal aneuploidies, whenever it gives a positive result as T21, T18 or T13 high risk, this pregnant woman would be referred to the NGS-NIPT or CVS/AC test for confirmation or diagnostics. From this standpoint, we believed it was reasonable to treat this T18 positive sample as a screen positive for trisomy because it would not be treated as a low-risk sample in a real world situation. Thus, the adjusted sensitivity and specificity of the dPCR-NIPT assay for screening overall fetal aneuploidies were 100% (36/36) and 95.12% (234/246), respectively (Table 5).

**Table 5:** Summary of the dPCR-NIPT assay results

			True aneuploidy statuses		dPCR-NIPT assay performance	
			+	−	Sens (%)	Spec (%)
dPCR-NIPT test results	T21^a^	+	25	7	100.00	97.28
		−	0	250		
	T18^a^	+	9	1	90.0	99.63
		−	1	271		
	T13	+	1	5	100.00%	98.22
		−	0	276		
dPCR-NIPT combined Screening results^*^		+	36	12	100.00	95.12
		−	0	234		

The test results of the dPCR-NIPT assay were compared to true aneuploid statuses for these samples. The top table with a clean background evaluated the dPCR-NIPT assay's T21, T18 and T13 performances, separately. The bottom table with a grey background demonstrated the dPCR-NIPT assay's overall performance as a screen tool by combining all the T21, T18 and T13 results. *Sens* sensitivity, *Spec* specificity

*One T18 positive sample was called as T21 by the dPCR-NIPT assay, this result was counted as T21 false positive and T18 false negative, separately, for the dPCR-NIPT assay's T21 and T18 evaluations. However, when the dPCR-NIPT assay acted as an intact test for screening aneuploid pregnancies, this T18 positive sample should be counted as a screen positive for trisomy

**Fig. 6:**
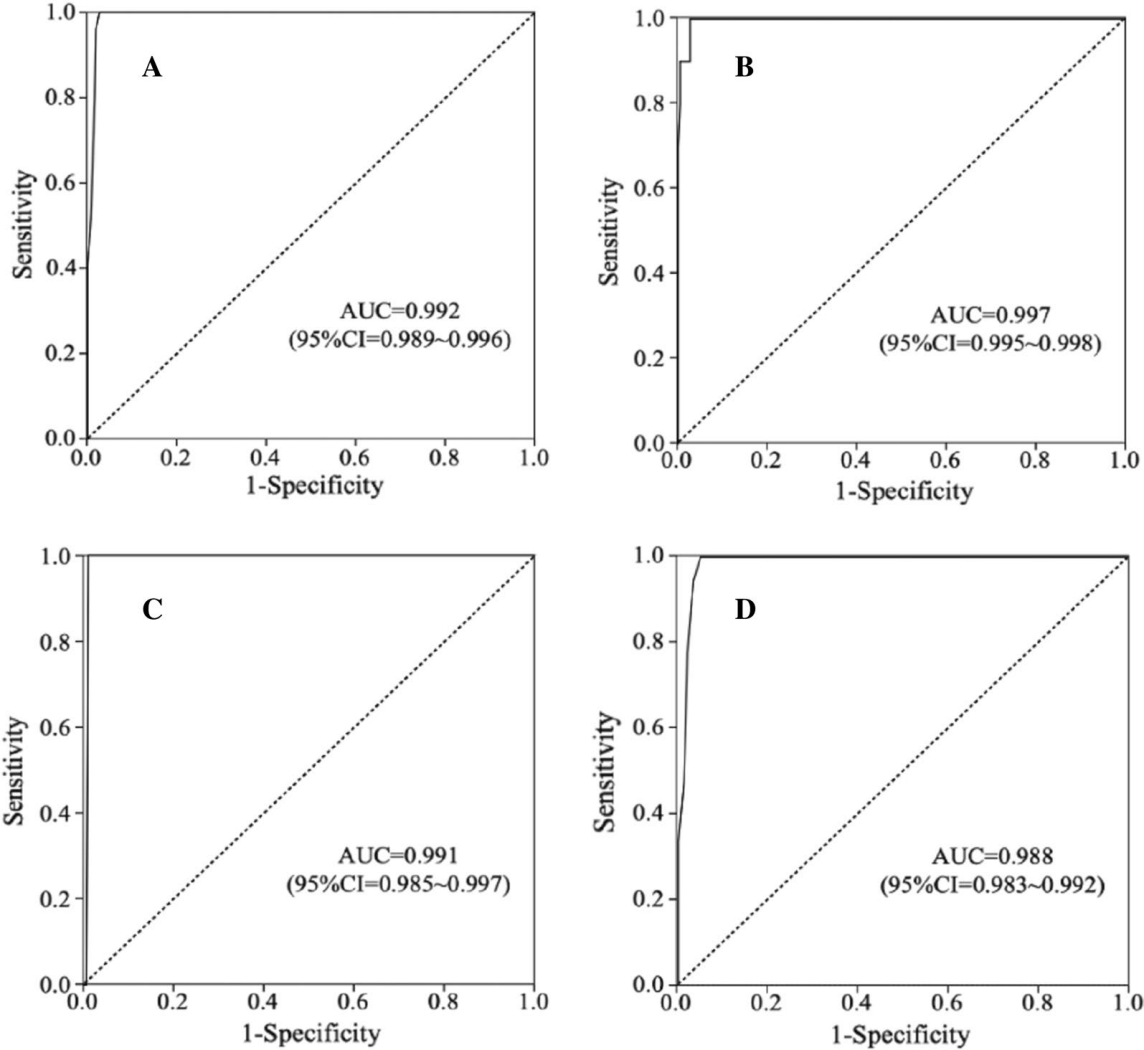
ROC curve analysis to assess the performance of the dPCR-NIPT assay over a range of Z-score cut-off values, both in a trisomy-specific manner and in a manner of combining all three trisomies as one screening test. The absolute value of a Z-score was used as a predictor of trisomies for samples exceeding the cut-off value of 1.65 for the number of high risk results. Panels** A**–**D** show the results of the T21, T18, T13, and the combined test, respectively, and the corresponding AUC value for each test is presented in each panel

A receiver operating characteristic (ROC) curve and the area under an ROC curve (AUC) are usually used to assess the performance of a diagnostic test [54], we plugged a ROC curve and calculated the AUC using the Z-score results of the 282 samples both in a trisomy-specific manner and in a manner of combining all three trisomies as one screening test (Fig. 6). The shapes of all the curves are very similar, except for T13, whose curve almost entirely parallels to the Y-axis due to only 1 true T13 positive sample tested. The AUC value is 0.992 (95% CI: 0.989–0.996) for the T21 test, 0.997 (95% CI: 0.985–0.998) for the T18 test, 0.991 (95% CI: 0.985–0.997) for the T13 test, and 0.988 (95% CI: 0.983–0.992) for the combined test, indicating that the dPCR-NIPT assay would be an extraordinary test method for screening fetal aneuploidies. The results of the ROC curve analyses also supported to set the Z-score cut-off value at ± 1.65 for this assay.We also directly compared the performances of the dPCR-NIPT assay to those of the SBT for 111 samples with test results from both methods. Of the 111 samples, 43, 66 and 2 were tested as high risk (risk rate ≥ 1/270), intermediate risk (1/270 > risk rate ≥ 1/1000) and low risk (risk rate < 1/1000) by the SBT, respectively. Of the 43 high risk and 66 intermediate risk samples, 5 (11.63%) and 3 (4.55%) were confirmed as T21 positive by CVS/AC tests, respectively, signifying the rest 38 (88.37%) high risk samples and the 63 (95.45%) intermediate risk samples were false positive to the SBT. Of the 2 low risk samples, 1 was true T21 positive and the other was true T18 positive according to the karyotypical tests, and both were counted as false negative for the SBT (SBT risk rate was 1/5,000 for the T21 sample and 1/1,163 for the T18 sample, respectively). Combining all above results of the 111 samples, the SBT showed a sensitivity of 80% (8/10) and a specificity of 0% (0/101) versus the true aneuploid results. However, of the same 111 samples, the dPCR-NIPT assay detected all the 10 (9 T21 and 1 T18) trisomies with 1 false positive each for T21, T18 and T13, and showed a sensitivity of 100% (10/10) and a specificity of 97% (98/101) compared to the true aneuploid results. According to this set of data, the dPCR-NIPT assay demonstrated a higher sensitivity and much better specificity than the SBT.

**Fig. 7:**
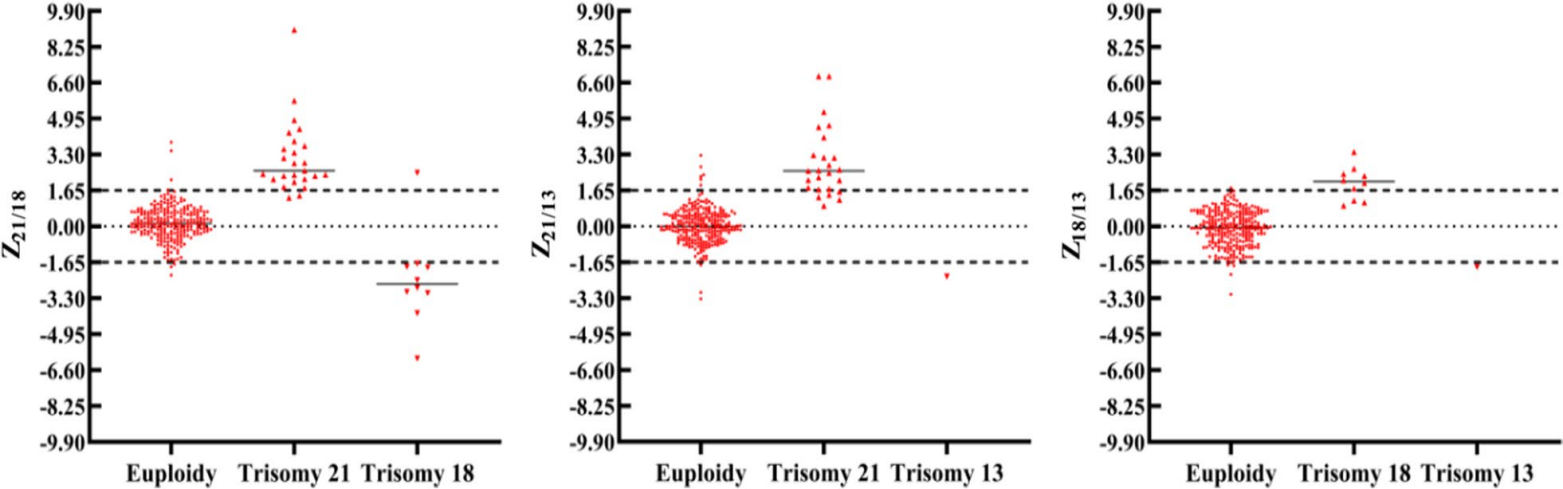
The Z-score distributions for Z_21/18_ (left panel), Z_21/13_ (middle panel), and Z_18/13_ (right panel) of the 282 samples were diagramed. In each panel the two horizontal dashed lines indicate the ± 1.65 Z-score cut-off, the short solid line represents the median Z-score for each Z-score group

For a screen test, the first priority is always the safety, i.e., making the positive detection rate as high as the technology allows while keeping the false positive rate at an acceptable level. Our results verified that the Z-score cut-off value of 1.65 and the calling algorithm (Table 4) applied would be appropriate with the sample volume available and the dPCR system used. When the 282 samples were sorted with their Z-scores, the negative samples were scattered between the Z-score cut-off lines of -1.65 and 1.65, while the trisomic positive samples were distributed beyond the two lines (Fig. 7). Of the 36 trisomic positive samples, 27 (18 T21, 8 T18 and 1 T13, 75.0%) had both corresponding Z-scores beyond the cut-off lines (≥ 1.65 or ≤ -1.65), while 9 (7 T21 and 2 T18, 25.0%) had one of the two corresponding Z-scores beyond the cut-off lines (Table 6). The latter 9 trisomic positives could be missed by the dPCR-NIPT assay if the calling algorithm would require both corresponding Z-scores beyond the cut-off lines. We hypothesized that the reasons why some true positive samples have only one of the two corresponding Z-scores beyond the cut-off lines or some low risk samples have two Z-scores with conflict calls might be related to the microheterogeneities of the cfDNA target sequences presented in these samples as mentioned above. With the current conditions presented for this study, the dPCR-NIPT assay still demonstrated a satisfactory performance in detection of T21, T18 and T13 in a single dPCR reaction with an overall sensitivity of 100% and specificity of 95%.

**Table 6:** Positive sample information and test results

Pos Cases	Sample information				Clinical results		The dPCR-NIPT results			
	GA (WKs)	MA (Yrs)	cfDNA (ng/uL)	cffDNA (%)	1st&2nd Tri-Scrn	CVS/AC Diagn	Z-scores		Final call	
							Z_21/18_	Z_21/13_	Z_18/13_	
1	12	26	0.30	12.03	Abn Ultr	T21	9.03	3.16	0.38	T21
2	13	28	0.48	11.97	Abn Ultr	T21	4.31	2.45	− 0.50	T21
3	12	44	0.24	10.22	Abn Ultr	T21	3.55	2.81	1.18	T21
4	13	28	0.42	10.41	Abn Ultr	T21	2.92	2.11	− 0.42	T21
5	12	29	0.22	9.83	Abn Ultr	T21	2.90	2.60	− 0.42	T21
6	13	27	0.32	7.11	Abn Ultr	T21	2.32	2.56	0.93	T21
7	13	30	0.48	11.35	Abn Ultr	T21	2.31	2.54	0.70	T21
8	12	20	0.35	11.18	Abn Ultr	T21	2.04	1.72	− 0.38	T21
9	12	19	0.40	13.00	Abn Ultr	T21	1.81	2.24	0.68	T21
10	12	27	0.35	ND	Abn Ultr	T21	1.76	1.77	0.61	T21
11	12	33	0.41	15.80	Abn Ultr	T21	3.91	1.58	− 0.72	T21
12	13	34	0.28	16.83	Abn Ultr	T21	3.14	1.33	− 1.52	T21
13	12	39	0.24	15.85	Abn Ultr	T21	2.56	1.44	− 0.27	T21
14	12	29	0.49	ND	Abn Ultr	T21	2.37	1.21	− 0.12	T21
15	12	42	0.43	7.29	Abn Ultr	T21	2.16	0.94	− 0.32	T21
16	19	37	0.40	14.07	Adv MA	T21	5.77	6.89	0.90	T21
17	19	25	0.32	16.92	SBT HR	T21	4.47	5.25	0.74	T21
18	16	33	0.28	13.39	SBT HR	T21	3.38	6.89	0.56	T21
19	13	31	0.39	7.45	SBT HR	T21	2.36	1.68	− 0.89	T21
20	17	22	0.29	9.02	SBT HR	T21	2.17	3.27	0.80	T21
21	18	28	0.23	14.23	SBT HR	T21	1.41	4.56	0.59	T21
22	17	33	0.35	6.80	SBT IR	T21	4.88	4.08	0.66	T21
23	19	30	0.34	13.38	SBT IR	T21	3.69	4.63	0.72	T21
24	13	31	0.51	6.98	SBT IR	T21	1.31	2.12	1.02	T21
25	15	23	0.53	9.69	NGS	T21	2.40	3.15	0.37	T21
**26**	**13**	**27**	**0.37**	**6.71**	**Abn Ultr**	**T18**	**2.46**	**2.29**	**0.95**	**T21**
27	12	27	0.20	ND	Abn Ultr	T18	− 6.07	− 1.20	3.41	T18
28	12	30	0.21	6.59	Abn Ultr	T18	− 1.89	1.03	2.65	T18
29	13	29	0.31	18.13	Abn Ultr	T18	− 3.99	0.20	2.40	T18
30	12	37	0.32	8.86	Abn Ultr	T18	− 2.82	− 0.17	2.31	T18
32	12	40	0.27	15.03	Abn Ultr	T18	− 1.71	0.50	2.00	T18
33	12	32	0.42	9.50	Abn Ultr	T18	− 3.01	− 1.38	1.74	T18
34	12	29	0.31	ND	Abn Ultr	T18	− 3.06	− 0.87	1.18	T18
35	13	28	0.34	8.87	Abn Ultr	T18	− 1.85	− 1.51	1.10	T18
31	17	23	0.35	17.38	NGS	T18	− 2.47	1.05	2.12	T18
36	12	25	0.60	ND	Abn Ultr	T13	− 1.42	− 2.31	− 1.86	T13

The sample information, clinical test results and the dPCR-NIPT results for the 36 trisomic positive samples are summarized. Sample 26 (shown in bold) is the T18 positive sample called as T21 positive by the dPCR-NIPT assay. *GA* gestational age, *WKs* weeks, *MA* maternal age, *Yrs* years, *cfDNA* cell free DNA, *cffDNA* cell free fetal DNA, *1st&2nd Tri-scrn*, first and second trimester screens; Diagn, diagnostics, *Abn Ultr* abnormal ultrasound, *SBT* serum biochemical test, *HR* high risk, *IR* intermediate risk, *NGS* next generation sequence

## Discussion

By testing the 283 clinical maternal plasma samples in a real clinical environment, we validated the dPCR-NIPT assay on aspects of testing performances, clinical operationality, and user friendliness. With existing dPCR system available on the market, we overcame the challenges of restricted sample volume available and the limited cffDNA fraction and cfDNA concentration presented in the maternal plasma samples by effectively incorporating cffDNA enrichment, specific cfDNA target amplifications and a multiplexed assay design into the dPCR-NIPT assay. The overall test results demonstrated that the dPCR-NIPT assay with a minimum of 18,000 droplets has a significantly statistical power to distinguish aneuploidies from euploid noises with a 95% confidence and is readily applied to screen pregnancies for fetal aneuploidies in clinical. This assay can be further improved in three areas, using a dPCR system with multiple fluorescent detection channels and minimum of 80,000 PCR partitions, increasing plasma sample volume to 4 mL, and continuously optimizing the testing reagents to increase accuracy and reduce variations. A dPCR system with at least 80,000 PCR partitions can increase the test confidence to 99.9% when estimated by Eq. 6 using the same parameters as described above but with a k = 3. Obviously, increasing sample volume can provide higher cfDNA target concentrations leading to a higher PDR to the assay, and further optimizing the testing reagents can enhance the test performances. In addition, upgrading these three areas will increase the test’s precision, which plays a critical role in the dPCR assay. However, even with the current format described in this study, the dPCR-NIPT assay is still a valuable test for screening fetal aneuploidies.

NGS-NIPT is a very powerful tool for screening fetal aneuploidies with high test accuracy [27–29], the main reason why it has not been widely used as a first-tier screen method is its relatively high price [32–35]. The CS has been proposed and used in some counties for purposes of increasing the positive detection rate and reducing the overall costs involved with the NGS assay. In this strategy, pregnant women are first screened by the SBT with a reduced risk cut-off, and then those with a risk rate ≥ 1/1000 were further tested by the NGS-NIPT for confirmations. Since the NGS-NIPT assay only tests the high and intermediate risk pregnancies funneled by the SBT, the positive detection rate of the existing CS is still fully determined by the less sensitive SBT. With a reduced risk cut-off of 1/1000, the SBT showed a higher positive detection rate with a range of 88.9–94.2% due to more samples were referred to the NGS-NIPT test, but also caused a much higher false positive rate with a range of 11.6–17.8%[32, 33, 55]. Fortunately, these SBT false positives were eventually balanced by the NGS-NIPT assay, thus the most significant benefit of this strategy is to dramatically reduce the number of invasive CVS/AC procedures crediting to the high specificity of the NGS-NIPT assay. Our data revealed that, even with the lowered risk cut-off of 1/1,000, the SBT still could not detect 1 T21 sample and 1 T18 positive sample, meanwhile, brought 63 additional false positive samples that needed to be rescreened by the NGS-NIPT.

Considering the dPCR-NIPT assay has a better sensitivity and specificity, if using it to replace the SBT in the existing CS, the upgraded version would be more effective with a higher positive detection rate and significantly less false positive samples to be resolved by the NGS-NIPT test. A major concern about to use the NGS-NIPT as the first-tier fetal aneuploidy screening test is lack of cost effectiveness due to its relatively higher price at an average of $570 per test with a range of $200-$1,100, while the SBT price is averaged at $53 with a range of $45-$77, depending on the countries [31–35]. If the price can be reduced to an average of $103 with a range of $61-$200 (depending on countries), the NGS-NIPT becomes cost-effective as the first-tier screen test when compared to the existing CS [32–35]. We estimate that the costs of the dPCR-NIPT assay is less than $100 and can even be close to the SBT price after the dPCR consumables being in a mass production. From the cost effectiveness and general performance points of view, the dPCR-NIPT assay provides an immediate solution for NIPT with a higher positive detection rate towards to the NGS-NIPT and a lower price close to the SBT. We would propose an upgraded version of CS for clinical application: all pregnant women are first screened by the dPCR-NIPT assay for trisomic (T21, T18 and T13) fetuses, the high risk ones are then rescreened by the NGS-NIPT test for confirmations, and finally the NGS confirmed high risk ones are diagnosed by the CVS/AC tests. To evaluate the cost effectiveness, we created a simulation situation to estimate and compare the costs for the three screening strategies (i.e., existing CS, the NGS-NIPT as first-tier screen strategy and the upgraded CS) with the following assumptions: 1) 10,000 samples to be tested by each strategy, 2) the average price, as mentioned above, for each of the three first-tier screen methods to be used, and 3) the average false positive rate of 15% for the SBT[32, 33, 55] and 4.88% for dPCR-NIPT to be used. In this simulation exercise, the upgraded CS had the lowest total costs and could save $107,980 (7.8%) and $4,422,980 (77.6%) when compared to the existing CS and the NGS-NIPT as the first-tier screen strategy, respectively (Table 7). The total cost difference between the existing CS and the upgraded CS seems marginal, however, the latter will provide substantial clinical benefits by enormously increasing the positive detection rate at a lower total cost. It should be mentioned that the turnaround time (TAT) for the existing CS and upgraded CS are comparable, since the NGS-NIPT test used in both CS for the high risk confirmation has a much longer TAT (above 20 h) and makes the time difference between the two primary screen methods negligible. From both the test performance and the cost effectiveness points of view, the dPCR-NIPT assay would be a better solution as a primary screen technology for screening fetal euploidies.

**Table 7:** Cost comparison of different strategies for screening fetal aneuploidies

Screen strategy^1^	Sample number	1st screen				2nd screen		Total cost ($)
		Method	$/Test^2^	Cost($)	HR case^3^	Method	Cost ($)	
Existing CS	10,000	STB	53	530,000	1500	NGS-NIPT	855,000	1,385,000
NGS-NIPT	10,000	NGS-NIPT	570	5,700,000	–	–	–	5,700,000
Upgraded CS	10,000	dPCR-NIPT	100	1,000,000	486	NGS-NIPT	277,020	1,277,020

*CS* contingent strategy, NGS-NIPT used as first-tier screen method; 2. $/test, the average price per test reported for STB and NGS-NIPT, the price for dPCR NIPT is estimated; 3. HR case, the high risk cases identified by the respective CS and the number is calculated based on the false positive rate of 15% for existing CS and 4.86% for the upgraded CS

## Conclusion

In this study, we evaluated the dPCR-NIPT assay’s non-clinical characteristics by verifying the cffDNA fraction enrichment efficiency, the PA uniformity for all multiplex cfDNA specific targets, the analytical sensitivity on artificial trisomic samples with low cffDNA fractions, and the sample quality control on minimum cfDNA concentration required. We validate the clinical performance for this assay by testing 283 plasma samples from high risk pregnancies and demonstrated that the sensitivity and specificity of the dPCR-NIPT assay is superior to those of the SBT. With a brief cost effectiveness analysis based current market prices, compared to the existing CS and the NGS-NIPT as the first-tier screen strategy, the upgraded CS, in which the dPCR-NIPT assay is used as a primary screen tool followed by the NGS-NIPT rescreen, is the most economical approach with improved sensitivity and specificity to screen pregnant women for fetal aneuploidies.

## Supplementary Information

**Additional file 1: Figure S1.** The structure of the microfluidic chip.** Figure S2.** The BioDigital-QING dPCRTM system.** Table S1.** Training set sample information.** Table S2.** Sequence information for primers and probes.** Table S3.** Estimation of number of droplet required for a dPCR reaction.

## Data Availability

All data are within the manuscript and its Additional file 1.

## Abbreviations

NGS: Next generation sequencing
NIPT: Non-invasive prenatal test
SBT: Serum biochemical tests
dPCR: Digital PCR
dPCR-NIPT: Digital PCR NIPT
cfDNA: Cell free DNA
cffDNA: Cell free fetal DNA
CS: Contingent strategy
PAPP-A: Plasma protein A
hCG: Human chorionic gonadotropin
AFP: Alpha-fetoprotein
uE3: Unconjugated estriol
CVS: Chorionic villus sampling
AC: Amniocentesis
NADT: Nucleic acid detection technologies
CCN: Chromosome copy number
BL: Baseline
CPM: Confined placental mosaicism
ROC: Receiver operating characteristic
TAT: Turnaround time
