# Supplementary material for: A dPCR-NIPT assay for detections of trisomies 21, 18 and 13 in a single-tube reaction-could it replace serum biochemical tests as a primary maternal plasma screening tool?
Source: J Transl Med. 2022 Jun 15;20:269. doi: 10.1186/s12967-022-03455-y (PMC9198625; doi:10.1186/s12967-022-03455-y)
Supplement: Supplementary file 1 — Additional file 1: Figure S1. The structure of the microfluidic chip. Figure S2. The BioDigital-QING dPCRTM system. Table S1. Training set sample information. Table S2. Sequence information for primers and probes. Table S3. Estimation of number of droplet required for a dPCR reaction. [file 12967_2022_3455_MOESM1_ESM.doc]

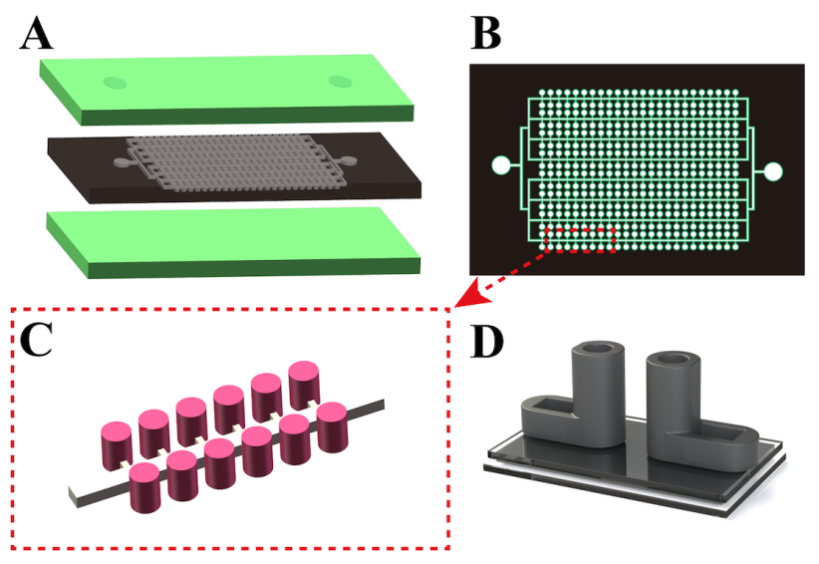


**Figure S1** The structure of the microfluidic chip. (A) diagram of the layered chip structure, (B) schematic of the microfluidic dPCR chip (31mm X 17mm) containing channels and reaction chambers, (C) the details of the channels and reaction chambers, (D) the physical appearance of the chip (35mm x 20mm).


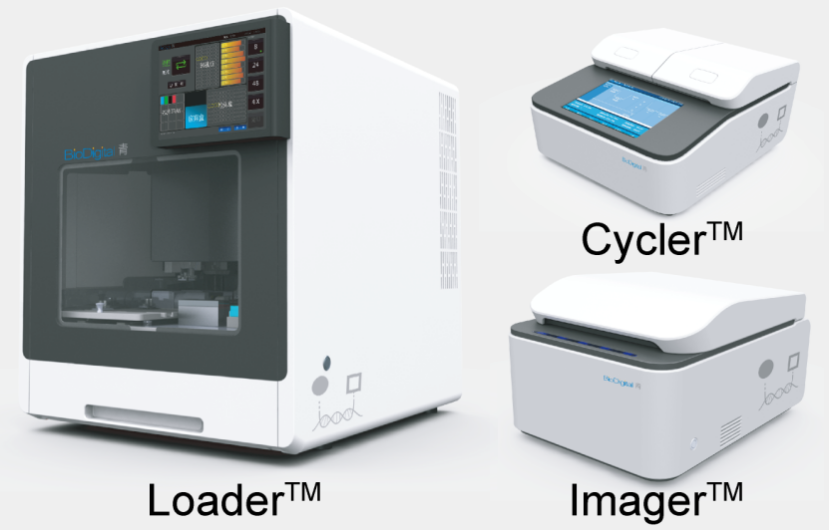


**Figure S2** The BioDigital-QING dPCR^TM^ system consists of three separate instruments. BioDigital-QING Loader^TM^, BioDigital-QING Cycler^TM^ and BioDigital-QING Imager^TM^ with a dimension (width X depth X height) of 530mm X 636mm X 636mm, 361mm X 476mm X 222mm, and 436mm X 637mm X 327mm, respectively.

**Table S1 Training Set Sample Information**

| **Clinical Samples Sorted By** | **T21** | **T18** | **T13** | **Normal** | **Total** |
| --- | --- | --- | --- | --- | --- |
| **Maternal Age (Years)** |  |  |  |  |  |
| ≤24 | 0 | 0 | 0 | 36 | 36 |
| 25-34 | 2 | 1 | 1 | 96 | 100 |
| ≥35 | 0 | 0 | 0 | 34 | 34 |
| **Gestational Age (Weeks)** |  |  |  |  |  |
| 12-15 | 0 | 0 | 0 | 12 | 12 |
| 16-20 | 2 | 1 | 1 | 118 | 122 |
| 21-25 | 0 | 0 | 0 | 28 | 28 |
| ≥26 | 0 | 0 | 0 | 8 | 8 |
| **Screen Method** |  |  |  |  |  |
| SBT HR (Cut-off at 1/270) | 1 | 0 | 0 | 37 | 38 |
| SBT IR (Cut-off at 1/271-1/1000) | 1 | 0 | 1 | 48 | 50 |
| Abnormal Ultrasound Results | 0 | 1 | 0 | 21 | 22 |
| Adv. Maternal Age (≥35 Years) | 0 | 0 | 0 | 33 | 33 |
| Direct NGS-NIPT | 0 | 0 | 0 | 27 | 27 |
|  |  |  |  |  |  |
| **Total** | **2** | **1** | **1** | **166** | **170** |
| Abbreviation: SBT, serum biochemical test; HR, high risk; IR, intermediate risk; Adv., advanced; NGS-NIPT, next generation sequence based noninvasive prenatal test | | | | | |

**Table S2 Sequence Information for Primers and Probes**

| **Sequence Set Number** | **Sequences (5’ to 3’)** | |
| --- | --- | --- |
| **Chromosome 21**  Set 1-FP  Set 1-RP  Set 1-PR  Set 2-FP  Set 2-RP  Set 2-PR  Set 3-FP  Set 3-RP  Set 3-PR  Set 4-FP  Set 4-RP  Set 4-PR  Set 5-FP  Set 5-RP  Set 5-PR  Set 6-FP  Set 6-RP  Set 6-PR  Set 7-FP  Set 7-RP  Set 7-PR  Set 8-FP  Set 8-RP  Set 8-PR  Set 9-FP  Set 9-RP  Set 9-PR  Set 10-FP  Set 10-RP  Set 10-PR | ACTGCAAGGACCAAGCACAGA  ACCAGAACGTTTGACTGGCCA  FAM- CACCTGGACACCAACCCTTCCCTGCGAGCC-BHQ1  CACTGCACACCAGTACTCAGACT  GGGTGAGAAGATCTTGTGGAGGA  FAM- TCCCCACTCCTCCGCCAGGAGTCTCCGGA-BHQ1  CCCAGCGCAAAGAAAGGCTGT  GTCACATTTGTGGGATGCCTGGT  FAM- AGGTGCAGGCCACGCTCATCACCCGCA -BHQ1  CGTGGCCTTCAGAACGAGGAT  ATACCCTCCACTGCCGACACA  FAM- ATGCAGGCCAGCCACTCCAGACCCGAGC -BHQ1  GGATGGCCAAAGGGAACCATCT  CCATGACCCAGGGCTCTCTT  FAM- AGCAGGTCACTGGACGCATGGCTCTGGAGC -BHQ1  GGGGTGCCTTCATGTCTAAGGA  TGGACAGCACTCAACCGTCCT  FAM- ACCAGTGGTGCTGAGCGTTTGCCTCGGAAGG-BHQ1  GTTAGGGACCACGAGCACTGAA  CCTCCCAGAACCACAAAGGTCT  FAM- TGGCAGGACGGTCCAGGGCCAGTCAACC -BHQ1  GATCAGTCTGTCATCAGAGTCCGT  CGCAAATGGATGTTCGGAGGCA  FAM- CCAGGTGGCCTGGCTGAGTCTGTCCACG -BHQ1  CCGGAAGTCCTCAAGTAGTCCA  GCTGCCTCCACCCAAGTTGA  FAM- CGAGCTGCCGTGGCACTGCTACAAAACCCC -BHQ1  CGAAAGAGGGTGGAAGCGTCT  CCACCCAGCCTATCTCTCAACA  FAM- CCCTTGCCTGCACACACCTGGTGCAGA -BHQ1 | |
| **Chromosome 18**  Set 1-FP  Set 1-RP  Set 1-PR  Set 2-FP  Set 2-RP  Set 2-PR  Set 3-FP  Set 3-RP  Set 3-PR  Set 4-FP  Set 4-RP  Set 4-PR  Set 5-FP  Set 5-RP  Set 5-PR  Set 6-FP  Set 6-RP  Set 6-PR  Set 7-FP  Set 7-RP  Set 7-PR  Set 8-FP  Set 8-RP  Set 8-PR  Set 9-FP  Set 9-RP  Set 9-PR  Set 10-FP  Set 10-RP  Set 10-PR | CCTGAGGGCATAAGCTCCTTGA  CCACAGATGTTGGATGGCGCT  VIC- TGTGTGGGTGTCCCCTGCAGTATCCAGCAG -BHQ1  CCGGACAAGTGAATGCCAAGGT  GCTTGACTCTGCTGGCCCTT  VIC- AGCGAGCGCGGACAGAGGCTGGGCTTCTAA-BHQ1  GCTGTCATAGGAGGCTTTCGTCA  AGTTTCCGGAAAGAAGGCCCT  VIC- AGGCCACTGAGAGCCCAAAACGTGCCACAGG -BHQ1  CCAAAGCCGGCACATGGTAGA  CCTCGCCCAAGTGTCATGCA  VIC- CAGGCATGCTCGGACCCATGTGCTTGGTGG -BHQ1  GCTTTGCTCAGACCAGTGGCT  GCACAACCTCGCCATTGCA  VIC- CAGAAGGCGGCATGCTCGGTCTTTGCAGCCT -BHQ1  CTCCACGCGGCGAACTTT  AGCTGTGTCCTCTTGATGCCAA  VIC- CGCGCATTCGGTGACCGGTCCCCAAACA -BHQ1  TGCCTTGTTGGACATTCTGAGGA  CACTCCAGCTTCAGTCCGTCA  VIC- TGGTGTCGGGTTGAAGGACACCTGGGAGCAG -BHQ1  GGCTACCAAAGAAGCAGGCAGT  GAGACACTTGCATCTCCGAGTCA  VIC- CTGCCGCTGGCCATGCAGCTTCTTGGTGG -BHQ1  AGGTGGGTTGCATGCCCT  GTCTGCCAATCAACAACCGCAA  VIC- AAACCAGCCTCCCAGGGAGCGTATGGCTGC -BHQ1  GACTATAGCCTGTCTCCTGGCA  GCTTAGCTGGAGAATGGCCCTA  VIC- CTGGTGCCCTCTCGATGCAGAGTCCTGCTGC -BHQ | |
| **Chromosome 13**  Set 1-FP  Set 1-RP  Set 1-PR  Set 2-FP  Set 2-RP  Set 2-PR  Set 3-FP  Set 3-RP  Set 3-PR  Set 4-FP  Set 4-RP  Set 4-PR  Set 5-FP  Set 5-RP  Set 5-PR  Set 6-FP  Set 6-RP  Set 6-PR  Set 7-FP  Set 7-RP  Set 7-PR  Set 8-FP  Set 8-RP  Set 8-PR  Set 9-FP  Set 9-RP  Set 9-PR  Set 10-FP  Set 10-RP  Set 10-PR | GTGTTACAGCCTGCATCGCGT  TGACCCTGCCTACGGCTCT  Cy5- CCCATGAGTCCAGAGCTGCAGCCACCCTGC -BHQ2  GCCCTAAATGCTGCACAGGCA  CGAGCATCCTTTCTATGAAGGCA  Cy5- TTGGGCGCATGTCTCATGGTGGCCAGCAGC -BHQ2  GGAAGGTGAGCGCTGTGAGAT  GCGAATCGGCTACAACACTGGA  Cy5- CCCACGTGACCCTACAGGTTCCAGGCTCAGA -BHQ2  GCCCTGGTTAGCCCTACCAT  ACAAGGACGGTCTACCGCCA  Cy5- TGCAGGAGGGCTGTTTTCTGTTCGCTGTGCC -BHQ2  GCCCTTCTGTGCCCTCGTATCA  CTACCTTGGTGCTGGGAGACA  Cy5- CCCTGCTTGGAGGAAACCCAACCACCCAGTG -BHQ2  AGGGAGATTGGTAGAGACGCA  ACCTCACAGCCATACGAATAGCT  Cy5- TGCCTCTTCGGGTCCCTGTTCTCCAGCCGA -BHQ2  AATTGAGGTGGAGGGTGTTGCA  CGTCCAGCGTCTCATGTGGAA  Cy5- CCAAGAAATGCAGGATTCGCTGCGGTGCTGC -BHQ2  TGTGATCTCCATGGACCTGGGT  ACATCTTGGCTGGGCAGGTT  Cy5- TGGGCACGGCACAAGGGCATTGCAGCAC -BHQ2  CCCAATCCAGTGACGCCCAA  CACATCTGACAGAATTCCCTCCCA  Cy5- CCCAGACCATGGTGCCAGGCGTGTTGG -BHQ2  CTGTCTAACTGGCCAGGACATCT  CCAGCCCAGAGAGCCGA  Cy5- ACCTCGCGTGGCCATCCAAGATGGAGCACC -BHQ2 | |
| Abbreviation: FP, forward primer; RP, reverse primer; PR, probe | |  |

**Table S3 Estimation of Number of Droplet Required for a dPCR Reaction**

| **PDR**  **(%)** | **cffDNA Fraction** | | | | | | |
| --- | --- | --- | --- | --- | --- | --- | --- |
|  | **1%** | **3%** | **5%** | **10%** | **20%** | **30%** | **40%** |
| 10 | 1473081 | 166949 | 61292 | 16080 | 4412 | 2144 | 1313 |
| 20 | 695537 | 78828 | 28940 | 7593 | 2084 | 1012 | 620 |
| 30 | 435143 | 49317 | 18105 | 4750 | 1304 | 634 | 388 |
| 40 | 303831 | 34435 | 12642 | 3317 | 910 | 443 | 271 |
| 50 | 223913 | 25377 | 9317 | 2445 | 671 | 326 | 200 |
| 60 | 169384 | 19197 | 7048 | 1849 | 508 | 247 | 151 |
| 70 | 128911 | 14610 | 5364 | 1408 | 387 | 188 | 115 |
| 80 | 96434 | 10930 | 4012 | 1053 | 289 | 141 | 86 |
| 90 | 67405 | 7640 | 2805 | 736 | 202 | 99 | 61 |
| The number of droplets required for a dPCR reaction for different PDR and cffDNA fraction combinations was estimated by using Equation 6 with a fixed standard deviation at 1.96. | | | | | | | |
